# Behavioral adjustment moderates the effect of neuroticism on brain volume relative to intracranial volume

**DOI:** 10.1111/jopy.12858

**Published:** 2023-06-13

**Authors:** Qinggang Yu, Stacey M. Schaefer, Richard J. Davidson, Shinobu Kitayama

**Affiliations:** 1 Department of Psychology, University of Michigan, Ann Arbor, Michigan, USA; 2 Center for Healthy Minds, University of Wisconsin Madison, Madison, Wisconsin, USA

**Keywords:** behavioral adjustment, brain volume, neuroticism

## Abstract

**Objective:**

The present study examined whether the effect of neuroticism on brain structure is moderated by behavioral adjustment.

**Background:**

Neuroticism is widely thought to be harmful to health. However, recent work using proinflammatory biomarkers showed that this effect depends on behavioral adjustment, the willingness and ability to adjust and cope with environmental contingencies, such as different opinions of others or unpredictable life situations. Here, we sought to extend this observation to “brain health” by testing total brain volume (TBV).

**Method:**

Using a community sample of 125 Americans, we examined structural magnetic resonance imaging of the brain and quantified TBV. We tested whether the effect of neuroticism on TBV was moderated by behavioral adjustment, net of intracranial volume, age, sex, educational achievement, and race.

**Results:**

Behavioral adjustment significantly moderated the effect of neuroticism on TBV, such that neuroticism was associated with lower TBV only when behavioral adjustment was low. There was no such effect when behavioral adjustment was high.

**Conclusion:**

The present findings suggest that neuroticism is not debilitating to those who constructively cope with stress. Implications are further discussed.

## 1 | INTRODUCTION

Neuroticism—a global trait defined by a propensity to experience various negative emotions (Costa & McCrae, 1992; Goldberg, 1993)—has been linked to poor mental and physical health outcomes (Clark et al., 1994; Friedman & Kern, 2014; Suls & Bunde, 2005). Moreover, neuroticism is associated with neural signatures of chronic stress and emotion dysregulation, including greater amygdala reactivity, decreased amygdala-prefrontal connectivity, and reduced gray matter (GM) volume (Forbes et al., 2014; Knutson et al., 2001; Ormel et al., 2013). However, neuroticism may not be uniformly “unhealthy,” as its effect depends on other psychological processes (Kitayama et al., 2018; Luchetti et al., 2014; Turiano et al., 2013). Prior work proposes that neuroticism is unhealthy primarily for those who are not able or willing to adjust their behaviors to environmental contingencies (Kitayama et al., 2018). Using an index of biological health risk (an amalgam of inflammatory and cardiovascular measures) as the outcome, the researchers observed that neuroticism predicts an increased biological health risk only for those relatively low in behavioral adjustment. The present study extends this work and tests whether behavioral adjustment moderates the previously documented inverse association between neuroticism and brain volume (Forbes et al., 2014; Knutson et al., 2001; Ormel et al., 2013).

### 1.1 | Neuroticism, health, and behavioral adjustment

Neuroticism is centrally defined by negative affectivity. Prior work has linked neuroticism to heightened stress reactivity and the propensity to experience anger, sadness, and disgust. (Costa & McCrae, 1992; Izard et al., 1993; Schneider, 2004). Therefore, neuroticism is considered a major risk for psychopathology, especially depression and anxiety disorders (Bienvenu et al., 2001; Clark et al., 1994). Neuroticism is also associated with poorer subjective health (Smith & MacKenzie, 2006; Suls & Bunde, 2005). This pattern has been corroborated by objective health indicators, such as body mass index (BMI) and cardiovascular mortality risk (Armon et al., 2013; Hagger-Johnson et al., 2012).

However, some inconsistencies exist in the literature, especially when health is objectively measured. For instance, in a study testing a large group of British participants (*N* = 321,456), neuroticism predicted reduced all-cause mortality (Gale et al., 2017). Another study found that neuroticism was not associated with poorer health, as assessed by proinflammatory markers, including C-reactive protein (CRP) and interleukin-6 (IL-6) (Luchetti et al., 2014). These inconsistent patterns may suggest that there exist important unmeasured variables that moderate the effect of neuroticism on health.

For instance, even though neuroticism predisposes individuals to experience more stress in response to situational demands and contingencies (Costa & McCrae, 1992; Schneider, 2004), such stress reactions can be either adaptive or maladaptive (Mendes et al., 2002) depending on one’s ability and willingness to adjust one’s behavior to the situations. This propensity to adjust one’s behavior to environmental contingencies is referred to as behavioral adjustment (Kitayama et al., 2018; Markus & Kitayama, 2003). For those who are more willing to adjust their behaviors to environmental contingencies (e.g., different opinions by others, difficult life situations), neuroticism and the associated stress reactivity may be functional because they are more likely to construe the potential threat as a challenge (Tomaka et al., 1993), which has salubrious health effects (Brosschot et al., 1998). However, neuroticism may have a negative impact on those who are not capable or unwilling to cope with them. Altogether, the above-noted inconsistencies in the association between neuroticism and health could be due, at least in part, to the failure of previous work to consider individual differences in behavioral adjustment.

Recent work tested community samples of both Americans and Japanese while assessing behavioral adjustment with a five-item scale (Kitayama et al., 2018). They found that for those high in behavioral adjustment, neuroticism is significantly associated with reduced biological health risks (assessed with proinflammatory cytokines [IL-6, CRP] and markers of cardiovascular malfunctioning [systolic blood pressure and the ratio of total-to-H DL cholesterol]). However, for those low in behavioral adjustment, neuroticism was associated with increased biological health risks. In another related study, Turiano et al. (2013) found that neuroticism was associated with lower levels of IL-6 among Americans who are high in conscientiousness. However, this finding proved elusive. Kitayama et al. (2018) used the same dataset and duplicated the conscientiousness x neuroticism interaction on IL-6 for Americans. However, this interaction was no longer significant when the summary index of biological health risk (used in their analysis of the neuroticism x behavioral adjustment interaction) was used. Moreover, regardless of the indices of biological health used, this interaction was negligible for the Japanese. More work is needed to assess the validity of the claim that conscientiousness moderates the health impact of neuroticism.

### 1.2 | Neuroticism and structural properties of the brain

The observed association between neuroticism (combined with low behavioral adjustment) and greater biological health risks has implications for neural processes. Specifically, individuals scoring high on neuroticism are more likely to perceive situations as stressful and react inadequately to stress. Prior evidence suggests that neuroticism can dysregulate the hypothalamic–pituitary–adrenal axis (Chrousos & Gold, 1992; Zobel et al., 2004), and the resulting chronic rise of cortisol can cause atrophy of the brain by speeding up the excitotoxic processes in the neurons (Sapolsky, 1994). Hence, it stands to reason that especially among those low in behavioral adjustment, neuroticism should be associated with reduced volume of the brain globally.

Prior studies found that neuroticism is inversely associated with the total brain volume controlling for the intracranial volume (Bjørnebekk et al., 2013; Knutson et al., 2001). One major drawback of the existent evidence is the lack of any measures of behavioral adjustment. Our theoretical analysis, consistent with the initial evidence (Kitayama et al., 2018), suggests that the inverse association between neuroticism and reduced total brain volume (Knutson et al., 2001) could be attenuated or even reversed for those high in behavioral adjustment. For this purpose, we assessed the total brain volume and tested whether it would depend on both neuroticism and behavioral adjustment. We also explored whether conscientiousness might also moderate the association between neuroticism and brain volume, given the previous finding of the moderating role of conscientiousness on the effect of neuroticism. Lastly, we also explored whether other Big Five traits moderate the link between neuroticism and brain volume. These analyses on the other Big Five traits are included in Supporting Information.

## 2 | METHODS

### 2.1 | Participants

We analyzed data from the Midlife in the United States (MIDUS; http://www.midus.wisc.edu) study. The participants were from the MIDUS “Refresher” cohort. The “Refresher” cohort included a national probability sample of 3577 participants, as well as a separate sample of 508 Black Americans from Milwaukee, WI. The study administered a series of questionnaires, including the measure of personality traits, which is of interest in the present study. One hundred thirty-eight participants from the “Refresher” cohort later participated in the “Refresher” Neuroscience study, which included various cognitive and emotional tasks as well as MRI brain scanning. Among these 138 participants, 127 underwent the MRI brain scanning because the rest of them did not meet the inclusion criteria for MRI scanning (e.g., no history of neurological disorders, no magnetic metal or medical devices in the body, no claustrophobia, ability to lie down on one’s back for two hours). For the remaining 127 participants, one was excluded due to missing survey data, and one was excluded due to left-handedness. Among the remaining 125 participants (58 males, 67 females), 78 were European Americans, 40 were African/Black Americans, 2 were Native Americans, 1 was Asian American, and 4 were of other races. The average age was 48.61 years (SD = 11.96), with ages ranging between 26 and 76 years. All participants provided informed consent before the study procedures. Survey data are publicly available from the ICPSR website (https://www.icpsr.umich.edu/web/ICPSR/series/203). Brain images are available from the MIDUS core team upon request (https://midus.wisc.edu/midus_neuro_data.php/).^1^

### 2.2 | Measures

Personality traits, including neuroticism, were measured using a 31-item adjective list (Rossi, 2001). Participants were asked to indicate how much each of the self-descriptive adjectives described themselves using a 4-point Likert scale (1—A lot, 2—Some, 3—Little, 4—Not at all). Neuroticism was assessed using 4 items: moody, worrying, nervous, and calm (reverse-coded). All items except the reversed-coded one were then reversed, so higher number indicates higher standing on the scale. The scores across the items were averaged. The mean neuroticism score of the participants in the present study was 2.18 (SD = 0.69). The reliability of the neuroticism scale was adequate (Cronbach’s alpha = 0.724) in the original MIDUS “Refresher” cohort. It was adequate among the participants of the current study as well (Cronbach’s alpha = 0.634).

The behavioral adjustment was measured using a 5-item adjustment scale (Kitayama et al., 2018). The items include “I usually follow the opinions of people I can respect,” “When many people have an opinion different from mine, I can adjust mine to theirs,” “When values held by others sound more reasonable, I can adjust my values to theirs,” “Once something has happened, I try to adjust myself to it because it is difficult to change it myself,” and “It is useless to try to change what is going to happen in life because it is impossible to predict it”. Participants rated each item on a 7-point Likert scale (1—Strongly disagree, 7—Strongly agree). The scores across the items were averaged. The mean behavioral adjustment score of the participants in the present study was 4.06 (SD = 0.97). The score was comparable to the one of a larger American sample used in Kitayama et al. (2018) (*M* = 4.11). The reliability of the behavioral adjustment scale was adequate (Cronbach’s alpha = 0.668).

### 2.3 | Covariates

We included several covariates in the current investigation to control for confounding effects. To ensure that our findings will be robust regardless of covariates used, we entered covariates in a stepwise fashion. The Step 1 covariate included the intracranial volume (see “Image Pre-processing and Measurement” section below for details) to control for individual difference in head size, as well as age (assessed at the time of scanning), sex (0—male, 1—female), and race (0—White Americans, 1—other racial groups). In Step 2 we included education, measured as the highest level of education completed, ranging from 1 (No school/some grade school) to 12 (PhD or other professional degree).

**FIGURE 1:**
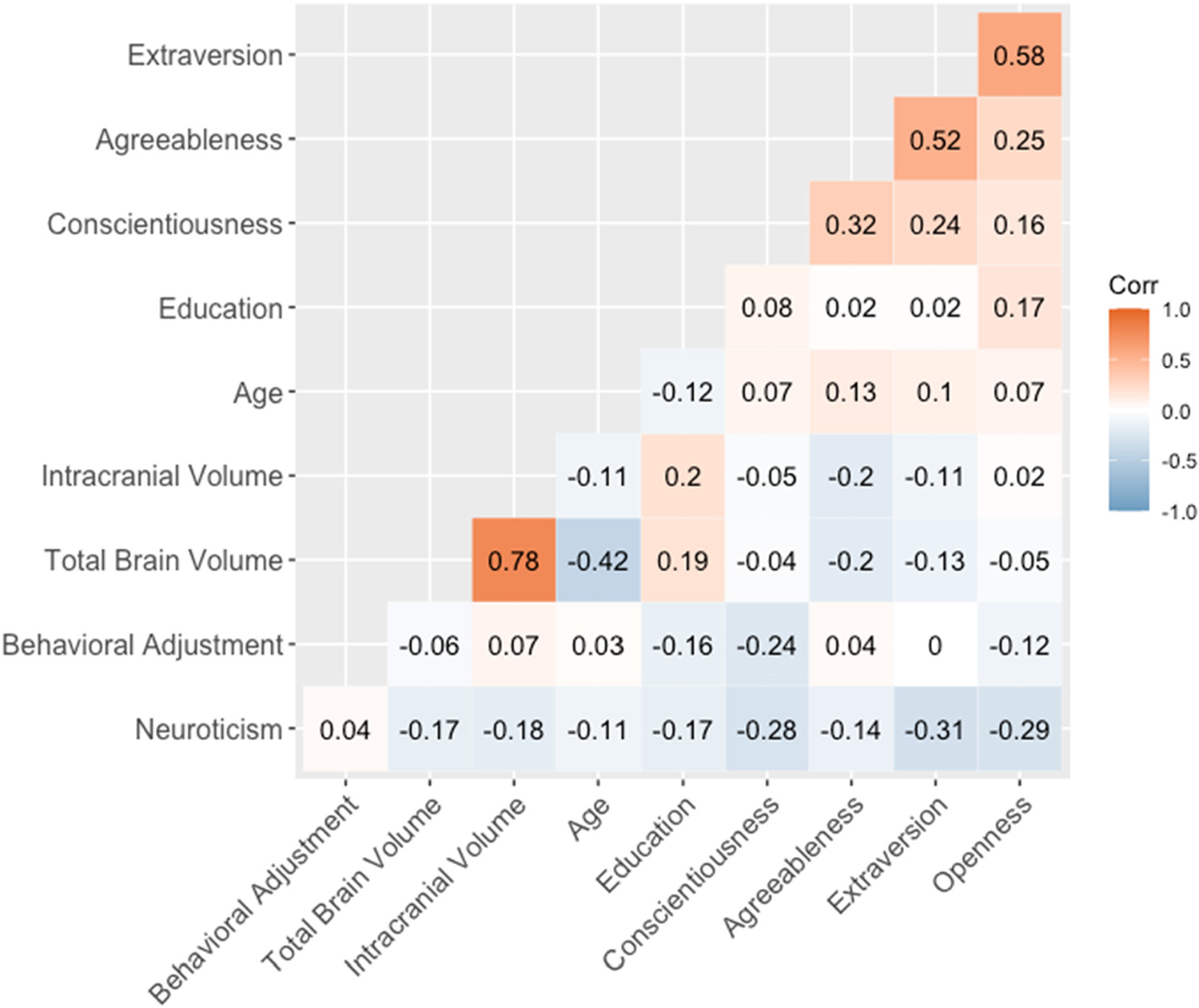
Pearson correlation coefficient of all continuous variables in the present study (*n* = 125).

In Step 2, we also controlled for conscientiousness, given that prior work suggests it may also moderate the effect of neuroticism. Participants indicated how much each of the self-descriptive adjectives described themselves using a four-point Likert scale (1—A lot, 2—Some, 3—Little, 4—Not at all). Conscientiousness was assessed using four items: Organized, Responsible, Hardworking, and Careless (R). All items except the reversed-coded one were then reversed, so higher numbers indicate greater conscientiousness. The scores across the items were averaged. The correlation matrix of all continuous variables in the present study, including all Big Five traits, is included in Figure 1.

### 2.4 | Image acquisition

Structural scans were acquired using a 3 T scanner (MR750 GE Healthcare, Waukesha, WI). A three-dimensional magnetization-prepared rapid gradient-echo sequence (Mugler & Brookeman, 1990) was used to acquire a T1-weighted anatomical image (repetition time = 8.2 ms, echo time = 3.2 ms, flip angle = 12°, field of view = 256 mm, 256 × 256 matrix, 160 1 mm axial slices per volume, inversion time = 450 ms).

### 2.5 | Image processing and measurement

Structural images were pre-processed and analyzed with the Statistical Parametric Mapping software (SPM; Wellcome Department of Cognitive Neurology, London, UK) using voxel-based morphometry (VBM) (Ashburner & Friston, 2000). VBM is an automated and unbiased technique to detect regionally specific and global differences in brain tissue composition. Each structural image was visually inspected for its orientation and origin point, and it was adjusted to match the template better if necessary. The images were then segmented into different tissue classes—gray matter (GM), white matter (WM), and cerebrospinal fluid (CSF) based on prior probability templates. The total GM, WM, and CSF volume were calculated by multiplying the total number of voxels of each tissue type by the voxel size. Total brain volume (TBV) was calculated by summing the total GM volume and total WM volume. Intracranial volume (ICV) was calculated by summing the volume of all the tissues. ICV was included in the model to control for individual difference in head size. In addition, examining TBV while controlling for ICV provides an estimate of brain atrophy relative to its maximal lifetime brain volume (Malone et al., 2015; Sargolzaei et al., 2015). The Pearson correlation between TBV and ICV was 0.78 in the current sample.

The images were also spatial normalized to conduct a whole-brain voxel-level analysis on neuroticism and behavioral adjustment. Details for the procedures and the results can be found in Supporting Information.

### 2.6 | Statistical analysis

We analyzed whether TBV would be associated with the level of neuroticism and whether behavioral adjustment would moderate this association. We performed multiple regression analysis by regressing TBV on neuroticism, behavioral adjustment, and their interaction. Covariates were included in the model in a stepwise fashion as mentioned above. All continuous variables in the regression model were centered. Categorical variables in the regression model were dummy coded. To further break down the interaction effect, we estimated the association between neuroticism and TBV (i.e., the predicted slope) at one standard deviation (SD) above and below the mean of behavioral adjustment, as well as at the mean of behavioral adjustment.

## 3 | RESULTS

### 3.1 | Total brain volume

**FIGURE 2:**
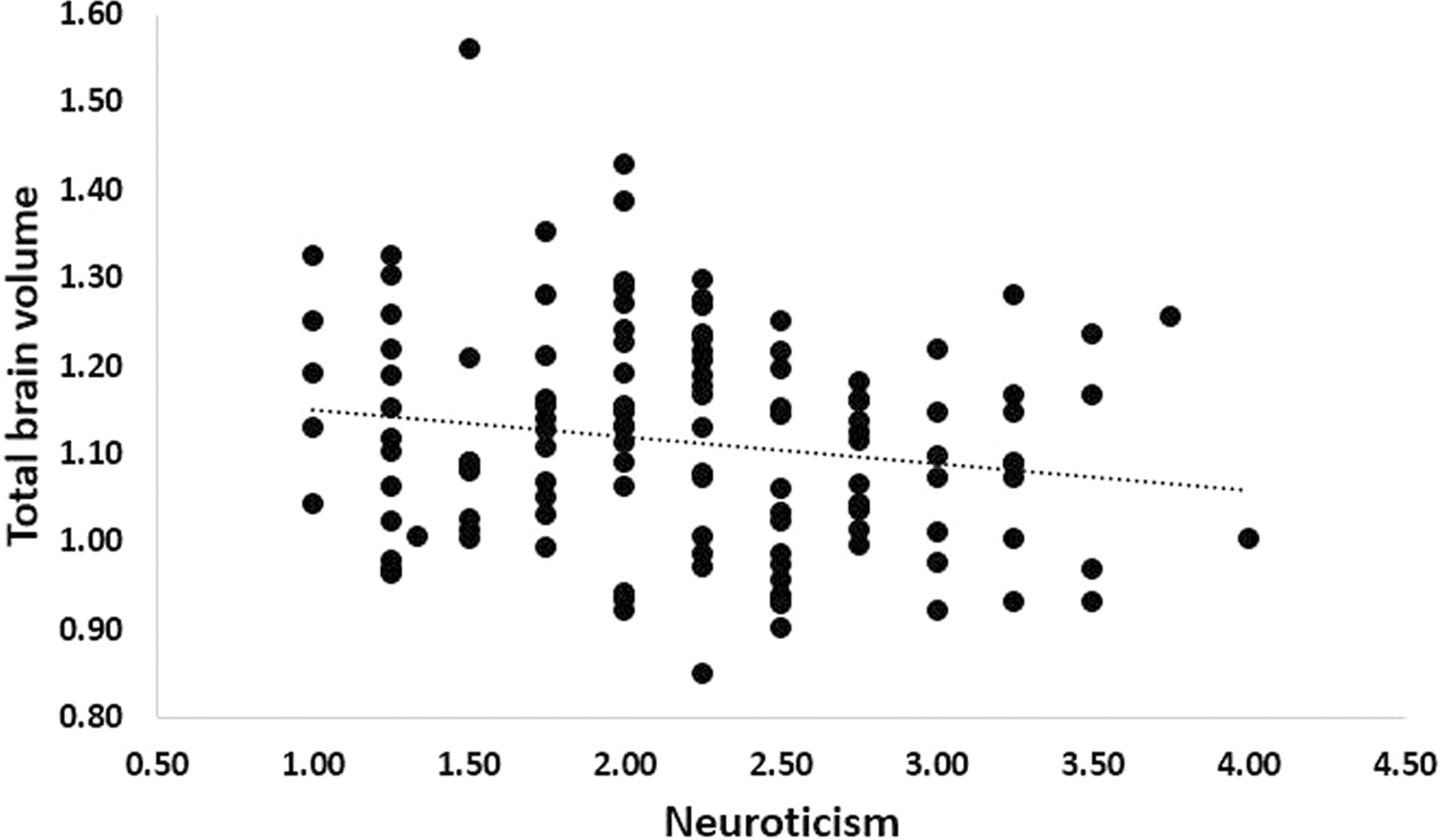
A scatterplot showing the negative correlation (zero-order) between neuroticism and total brain volume (in liters).

We found that the association between neuroticism and TBV was not statistically significant, although it was in the negative direction (Figure 2). This was the case without covariates (*b* = −0.031, *t*(121) = −1.979, *p* = 0.050); when controlling for age, sex, race, and ICV (*b* = −0.015, *t*(117) = −1.773, *p* = 0.079); when additionally controlling for education and conscientiousness (*b* = −0.017, *t*(115) = −1.916, *p* = 0.058).

**FIGURE 3:**
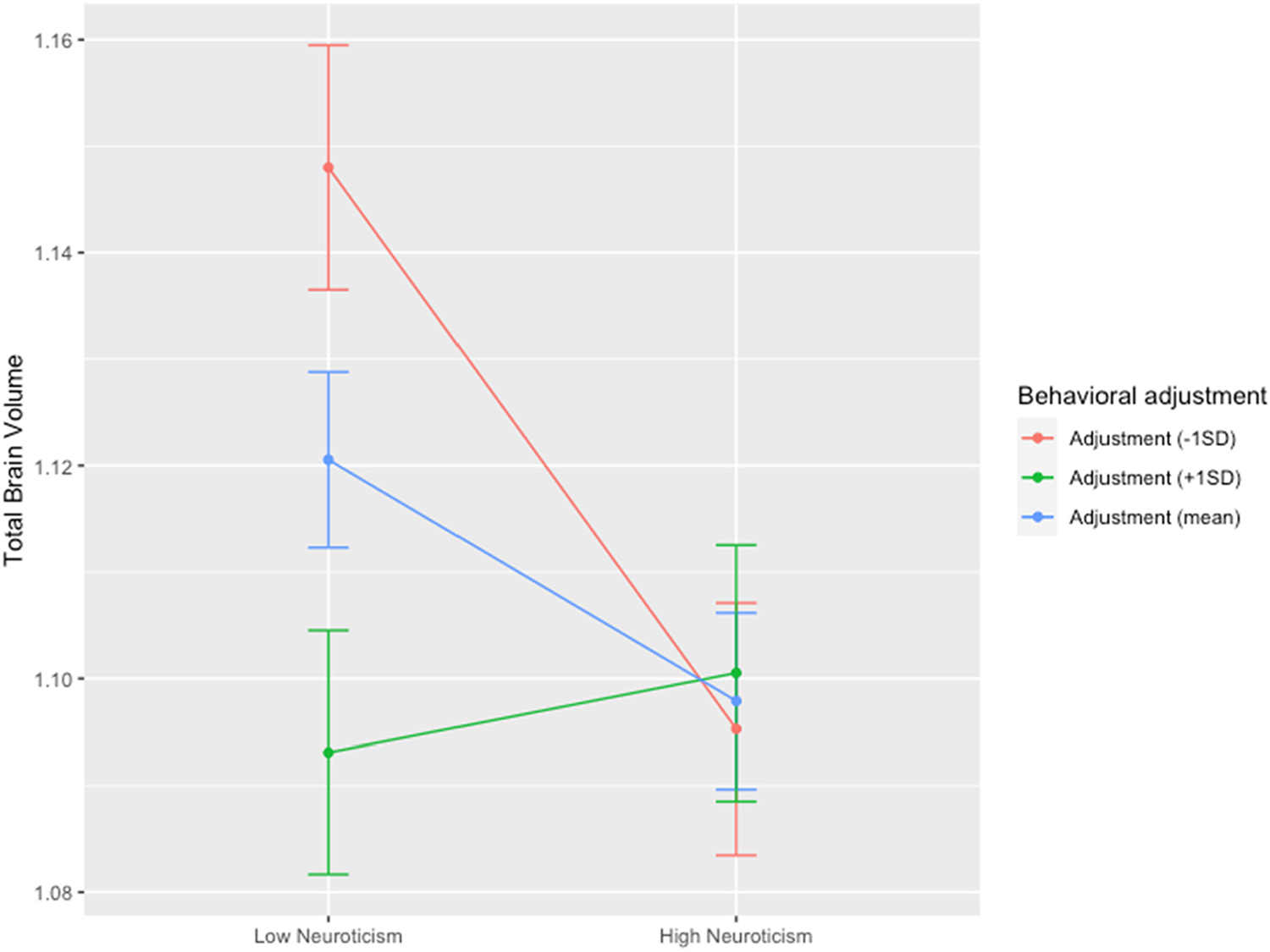
Mean value of total brain volume (in liters) as a function of neuroticism (±1 SD around the mean) and behavioral adjustment (at the mean and ±1 SD around the mean), after adjusting for covariates.

Importantly, however, the interaction term between neuroticism and behavioral adjustment was significant without covariates (*b* = 0.050, *t*(121) = 3.063, *p* = 0.003); when also controlling for age, sex, race, and ICV (*b* = 0.023, *t*(117) = 2.654, *p* = 0.009); when additionally controlling for education and conscientiousness (*b* = 0.022, *t*(115) = 2.547, *p* = 0.012). Subsequent simple slope tests (based on the model with all the covariates) indicated that neuroticism predicted less TBV when behavioral adjustment was lower (one SD below the mean), *b* = −0.038, 95% confidence interval (CI) = [−0.062, −0.014]. This effect, however, disappeared when behavioral adjustment was higher (one SD above the mean), *b* = 0.005, 95% CI = [−0.019, 0.029]. This effect was also negligible at the mean level of behavioral adjustment, *b* = −0.016, 95% CI = [−0.033, 0.001] (Figure 3).

In this analysis, we additionally found that behavioral adjustment predicted reduced TBV. This pattern was significant when all covariates were included in the model (*b* = −0.013, *t*(115*)* = −2.131, *p* = 0.035), though it was not significant without covariates (*b* = −0.004, *t*(121) = −0.335, *p* = 0.738) or only controlling for age, sex, race, and ICV (*b* = −0.011, *t*(117) = −1.973, *p* = 0.051). We will return to this effect in the discussion section. Detailed statistics of the regression can be found in Table 1.

## 4 | DISCUSSION

Prior work has shown reliable negative associations between neuroticism and the volume for either the entire brain (Knutson et al., 2001) or specific regions (Bjørnebekk et al., 2013; DeYoung et al., 2010; Riccelli et al., 2017; Wright et al., 2006). The central contribution of our work was to extend this work and show that behavioral adjustment significantly moderates the inverse association between neuroticism and the total brain volume. Behavioral adjustment refers to an individual difference variable implying the willingness and capacity of adjusting one’s behavior to cope with environmental contingencies. Our data indicate that neuroticism is significantly correlated with a smaller total brain volume for those low in behavioral adjustment, but this association is not significant for those high in behavioral adjustment.

Our data are consistent with the hypothesis that neuroticism sensitizes people to perceive threat cues and experience stress. For those lacking the ability or willingness to adjust one’s behavior to cope with situational demands, neuroticism may magnify the stress, thereby deregulating the hypothalamic–pituitary–adrenal axis (Chrousos & Gold, 1992; Zobel et al., 2004). The resulting rise of cortisol could be associated with atrophy of brain tissue. For those who are both able and willing to adjust their behaviors to cope with various situations, physiological pathways for regulating stress could remain intact, which attenuates the link between neuroticism and potential neurodegeneration. It is unclear whether the moderating effect of behavioral adjustment is localized to certain regions. Future work should further examine whether the moderating effect of behavioral adjustment (and other psychological processes) is localized to brain regions uniquely linked to neuroticism, or it is extended across the brain.

The present finding is also consistent with the idea of “healthy neuroticism” (Friedman, 2000). Instead of compromising biological health, neuroticism is sometimes associated with better health through healthy behaviors such as reduced smoking and reduced alcohol consumption (Turiano et al., 2012; Weston & Jackson, 2015). Most previous studies have focused on the interactive effect of neuroticism and other personality traits, such as conscientiousness, in examining “healthy neuroticism”. The present study suggests that another critical factor that may make neuroticism “healthy” is behavioral adjustment. This finding has various clinical implications. For instance, in psychotherapies that aim to address neuroticism (and associated stress and anxiety), it may be instrumental to encourage the practice of behavioral adjustment in the face of uncontrollable life situations.

One unexpected finding from our analysis came from the main effect of behavioral adjustment. The total brain volume was smaller (rather than larger) for those high in behavioral adjustment. This finding might be surprising since behavioral adjustment would appear adaptive, especially when combined with high neuroticism. The present study examined a sample of Americans. Behavioral adjustment may be incongruous with the cultural norms of individualism (Morling et al., 2002; Tsai et al., 2007). It is, therefore, possible that the resulting cultural mismatch made the act of behavioral adjustment stressful and taxing to many Americans, and consequently, it might have negative impacts on the brain. Nevertheless, this analysis was exploratory in nature and our data were cross-sectional. It is thus critical to empirically test this possibility in future work. It will also be informative to examine the effect of behavioral adjustment in interdependent cultures, where behavioral adjustment is more common and encouraged.

Some limitations of the present study must be acknowledged. First, the correlational nature of the present data prevents us from concluding on the directionality between neuroticism and the structural features of the brain. While we have argued that neuroticism and behavioral adjustment lead to changes in the brain, it is possible that preexisting patterns in the brain determine the level of neuroticism and behavioral adjustment. A longitudinal extension of the present study will be informative. Second, the present study used an abbreviated four-item scale of neuroticism. Previous studies have identified unique structural correlates of different facets of neuroticism (Bjørnebekk et al., 2013). Future work should use an extended measure of neuroticism and examine which aspect of neuroticism the behavioral adjustment is most likely to act upon. Third, earlier data by Kitayama et al. (2018) showed that neuroticism is associated with better biological health for Japanese. They found that the positive health effect of neuroticism is due to the high levels of behavioral adjustment of the Japanese. Future work must test whether analogous effects of neuroticism could be observed for brain volume.

These limitations notwithstanding, our work presents the first evidence that the neural correlate of neuroticism is modulated by how the individual responds and copes with environmental contingencies by adjusting their behavior. This work suggests that personality neuroscience (DeYoung, 2010) should carefully consider how other psychological factors may augment or attenuate the link between personality traits and brain structures (or functions).

## Supplementary Material

Supporting Information

## Figures and Tables

**TABLE 1:** Regression coefficients of models predicting total brain volume (TBV) as a function of neuroticism (N) and behavioral adjustment (BA).

	Model 1				Model 2				Model 3			
	*B*	Std. *B*	SE	*p*	*B*	Std. *B*	SE	*p*	*B*	Std. *B*	SE	*p*
N	−0.031	−0.169	0.015	0.050	−0.015	−0.080	0.008	0.079	−0.017	−0.091	0.009	0.058
BA	−0.004	−0.030	0.011	0.738	−0.011	−0.088	0.006	0.051	−0.013	−0.100	0.006	0.035
N×BA	0.050	0.271	0.016	0.003	0.023	0.124	0.009	0.009	0.022	0.121	0.009	0.012
Age					−0.003	−0.336	0.0004	<0.001	−0.004	−0.341	0.0004	<0.001
Sex					−0.022	−0.175	0.013	0.101	−0.020	−0.156	0.014	0.152
Race					−0.033	−0.267	0.012	0.006	−0.035	−0.285	0.012	0.004
ICV					0.472	0.644	0.040	<0.001	0.479	0.653	0.042	<0.001
Education									−0.002	−0.044	0.002	0.363
Conscientious									−0.005	−0.019	0.012	0.702
*R* ^2^	0.102				0.771				0.773			

## Footnotes

1 The present research and the analytic plan were not formally preregistered.
