# Supplementary material for: Behavioral adjustment moderates the effect of neuroticism on brain volume relative to intracranial volume
Source: J Pers. Author manuscript; Available in PMC 2024 Aug 1. (PMC10716358; doi:10.1111/jopy.12858)
Supplement: Supporting Information [file NIHMS1914836-supplement-Supporting_Information.docx]

**Supplementary Materials**

**Behavioral Adjustment Moderates the Effect of Neuroticism on Brain Volume Relative to Intracranial Volume**

**Qinggang Yu^1^**

**Stacey M. Schaefer^2^**

**Richard J. Davidson^2^**

**Shinobu Kitayama^1^**

**^1^University of Michigan**

**^2^University of Wisconsin Madison**

**A. Voxel-Level Whole-Brain Analysis and ROI Analysis**

In the main manuscript, we examined the total brain volume as a function of neuroticism, behavioral adjustment, and their interaction. Here, we examined whether the effects are localized to certain brain regions.

**Image Processing and Measurement**

After the structural brain images were segmented to different tissue classes, the “Diffeomorphic Anatomical Registration through Exponentiated Lie” (DARTEL) algorithm (Ashburner, 2007) was used to create a sample-specific template of GM. This template was then affine-registered to the Montreal Neurological Institute (MNI) space. All the segmented GM images were nonlinearly warped to match the space of this template. Modulation was performed to retain the original GM volume by multiplying the warped tissue probability map by the Jacobian determinant of the warp. Lastly, the modulated images were smoothed with a 10-mm full-width half-maximum Gaussian kernel.

To analyze the association between neuroticism and regional GM volume, we conducted whole-brain voxel-level analysis in SPM using the framework of general linear model on the pre-processed GM images. All voxels with a GM value smaller than 0.05 (value ranges from 0 to 1) were excluded to retain only the homogenous voxels. Nonstationary cluster extent correction was applied to correct for nonisotropic smoothness of VBM data (Hayasaka & Nichols, 2004). We used the multiple regression design with neuroticism as the predictor, while controlling for the covariates. The voxel-level threshold (height threshold) was set at *p* < .0001 (uncorrected). The cluster-level threshold of significance was then set at *p* < .05 family-wise error (FWE) corrected.

**Whole-Brain Voxel-Level Results**

The whole-brain voxel-level analysis showed that neuroticism was significantly correlated with the GM volume in the DLPFC (Figure S1) controlling for age, sex, race, education, conscientiousness, and ICV. Using the whole-brain voxel-level analysis, we found that neuroticism predicted reduced GM volume at a cluster within the left DLPFC (Peak voxel: MNI coordinate x = -22, y = 56, z = 28; Z value = 4.27; Cluster size k = 523; *p* = .026, FWE-corrected). In addition, this whole-brain analysis identified a cluster of voxels in the right visual association area (Peak voxel: MNI coordinate x = 21, y = -84, z = 20; Z value = 4.20; Cluster size k = 69; *p* = .226, FWE-corrected) negatively correlated with neuroticism. This cluster, however, did not survive the FWE correction. Hence, no further interpretation is made. See Figure S2 for the results of the whole-brain analysis. No region emerged as showing a positive association with neuroticism even when we loosened the voxel-level threshold to *p* < .001 (uncorrected).

We subsequently tested whether the inverse association between neuroticism and DLPFC GM volume was moderated by behavioral adjustment. We extracted the mean GM volume of the DLPFC. To avoid the issue of nonindependence in MRI analysis, we defined an independent anatomical region of interest (ROI), rather than using the peak cluster volume identified from the analysis above. We used Brodmann area 46 (BA 46) as the ROI (Figure S3), which maps onto the DLPFC in human brain (Petrides & Pandya, 1999). The ROI was constructed using the WFU PickAtlas toolbox (Maldjian et al., 2003), dilated with a factor of 1. We regressed the GM volume of BA 46 on neuroticism, behavioral adjustment, their interaction term, controlling for age, sex, race, education, conscientiousness, and ICV.

We found that the interaction between neuroticism and behavioral adjustment was not statistically significant, *b* = .002, *t*(115) = 0.588, *p* = .558. Although we observed that the negative effect of neuroticism on the GM volume at the left DLPFC was stronger when behavioral adjustment was low, this pattern should be interpreted with caution given the non-significant interaction effect.

Lastly, we performed an exploratory whole-brain voxel-level analysis testing whether any brain region shows a neuroticism x behavioral adjustment interaction. The procedures were the same as the analysis above testing neuroticism. The multiple regression design was used with neuroticism, behavioral adjustment, and the interaction term as the predictor while controlling for age, sex, race, education, conscientiousness, and ICV. However, this analysis did not identify any cluster that achieved the threshold of significance, even loosening the height threshold to *p* < .001 (uncorrected).


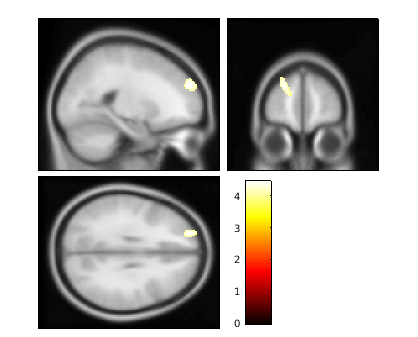


Fig. S1. Cluster at left dorsolateral prefrontal cortex (DLPFC) that shows significant negative correlation with neuroticism. Results are superimposed on the slices of a T1-weighted mean-image in sagittal (top left), coronal (top right), and axial (bottom left) view. The color bar shows the corresponding Z-scores.


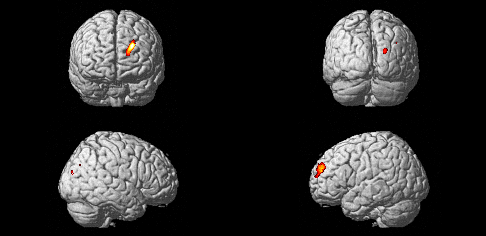


Fig. S2. Regions in which the GM volume was negatively correlated with neuroticism, shown in SPM render-style brain.


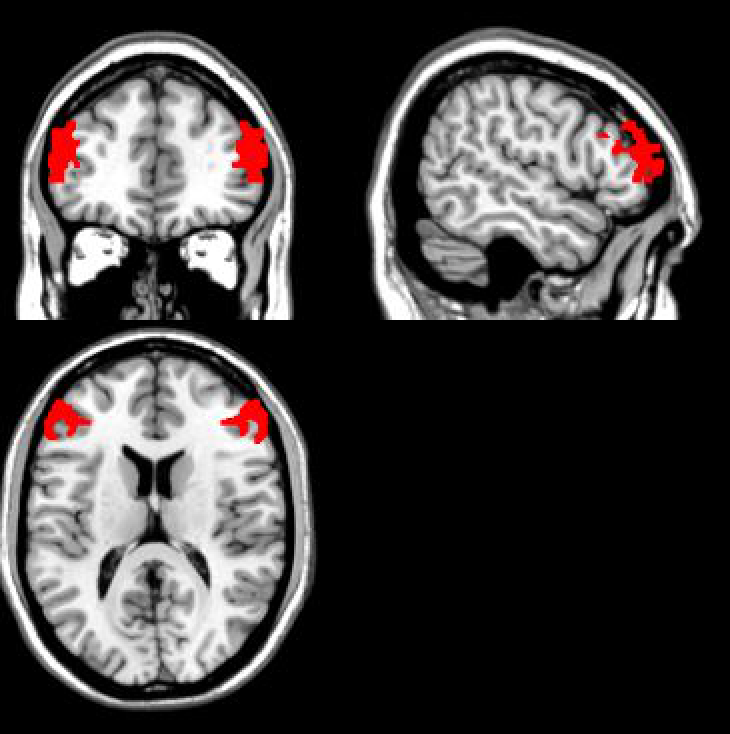


Fig. S3. The DLPFC region of interest (ROI) for the present study from a coronal (top left), sagittal (top right), and axial (bottom left) view. The ROI was created by combining the left and right Brodmann area 46.

**B. Other Exploratory Analyses**

We carried out two sets of exploratory analysis testing (1) the moderating effect of other Big 5 traits on the link between neuroticism and TBV, and (2) the effect of neuroticism when neuroticism was measured using an expanded set of items.

**The moderating effect of other Big 5 traits**

In the first set of analysis, we examined whether other Big 5 traits moderate the relationship between neuroticism and the TBV. The measure of neuroticism and conscientiousness was described in the main manuscript. Extraversion was assessed using five items: outgoing, friendly, lively, active and talkative. Openness to experience was assessed using seven items: creative, imaginative, intelligent, curious, broad-minded, sophisticated, adventurous. Agreeableness was assessed using five items: helpful, warm, caring, softhearted, sympathetic. Participants were asked to indicate how much each of the self-descriptive adjectives described themselves using a 4-point Likert scale (1 – A lot, 2 – Some, 3 – Little, 4 – Not at all). All items were then reversed, so higher number indicates higher standing on the scale.

We applied the same multiple regression method and used the same sets of covariates (age, sex, race, ICV, education) as in the main analysis. We found no evidence that conscientiousness was associated with TBV (*p* = .565), and neither was extraversion (*p* = .967), openness (*p* = .717), or agreeableness (*p* = .838). Moreover, we found no evidence of an interactive effect between neuroticism and conscientiousness on TBV (*p* = .457), and neither was extraversion (*p* = .970), openness (*p* = .317), or agreeableness (*p* = .380).

**Testing the effect of neuroticism using a composite measure**

In the second set of analysis, we expanded the items that we used to measure neuroticism of the participants. Traditionally, a more extensive measure of neuroticism (e.g., NEO Personality Inventory) usually consists of items that assess different facets of neuroticism: anxiety, anger hostility, depression, self-consciousness, impulsiveness, and vulnerability (Costa & McCrae, 1992). Hence, we adapted scales from the MIDUS study that measure constructs highly similar to some of the above facets.

In particular, we used *stress reactivity* and *aggression* from the Multidimensional Personality Questionnaire. Stress reactivity was measured using three items: “My mood often goes up and down”, “I sometimes get myself into a state of tension and turmoil as I think of the day's events”, and “Minor setbacks sometimes irritate me too much”. This measure is conceptually similar to vulnerability, which describes one’s susceptibility to stress. Aggression was measured using four items: “When I get angry I am often ready to hit someone”, “Sometimes I seem to enjoy hurting someone by saying something mean”, “When people insult me, I try to get even”, and “Sometimes I just like to hit someone”. This measure is thus conceptually similar to anger hostility, which describes one’s tendency to experience anger and frustration. Participants were asked to indicate how much each of the items described themselves using a 4-point Likert scale (1 – True of you, 2 – Somewhat true, 3 – Somewhat false, 4 – False). All items were then reversed, so higher number indicates higher standing on the scale. We then converted neuroticism, stress reactivity, and aggression to Z score, and they were averaged to create a composite measure of neuroticism.

Using the same multiple regression methods as in the main analysis, we found that there was a significant interaction between the composite measure of neuroticism and behavioral adjustment on TBV. This was the case when controlling for age, sex, race, and ICV (*b* = .124, *t*(117*)* = 2.386, *p* = .019); and when also controlling for education and conscientiousness (*b* = .122, *t*(115) = 2.334, *p* = .021).

**References**

Ashburner, J. (2007). A fast diffeomorphic image registration algorithm. *NeuroImage*, *38*(1), 95–113. https://doi.org/10.1016/j.neuroimage.2007.07.007

Costa, P. T., & McCrae, R. R. (1992). Four ways five factors are basic. *Personality and Individual Differences*, *13*(6), 653–665. https://doi.org/10.1016/0191-8869(92)90236-I

Maldjian, J. A., Laurienti, P. J., Kraft, R. A., & Burdette, J. H. (2003). An automated method for neuroanatomic and cytoarchitectonic atlas-based interrogation of fMRI data sets. *NeuroImage*, *19*(3), 1233–1239. https://doi.org/10.1016/S1053-8119(03)00169-1

Petrides, M., & Pandya, D. N. (1999). Dorsolateral prefrontal cortex: Comparative cytoarchitectonic analysis in the human and the macaque brain and corticocortical connection patterns. *European Journal of Neuroscience*, *11*(3), 1011–1036. https://doi.org/10.1046/j.1460-9568.1999.00518.x
